# An outlook on livestock welfare conditions in African communities — A review

**DOI:** 10.5713/ajas.19.0282

**Published:** 2019-07-01

**Authors:** Yonela Zifikile Njisane, Felicitas Esnart Mukumbo, Voster Muchenje

**Affiliations:** 1 Department of Livestock and Pasture Science, University of Fort Hare, P. Bag X1314, Alice 5700, South Africa; 2 Risk and Vulnerability Research Centre, University of Fort Hare, P. Bag X1314, Alice 5700, South Africa

**Keywords:** Livestock Wellbeing, Cultural Practices, Developing World, Food Security, Production Systems

## Abstract

A significant proportion of the African continent is conducive for animal agricultural production, due to its historical experience and available resources to accommodate and nurture various indigenous and exotic animal species and breeds. With food security being a global challenge, animal products can play an important role as nutrient dense food sources in human diets, particularly in Africa. However, this does not seem to reach its full potential in practice, due to numerous reasons that have not been adequately addressed. Animal welfare reservations can be highlighted as one of the major contributing factors to the curbed progress. The consequences have been scientifically proven to affect product quality and market access. However, in the African community, the concept of animal welfare has not been fully embraced. While there are international animal welfare standards in the developed world, there are inherent factors that hinder adoption of such initiatives in most developing regions, particularly among communal farmers. These include cultural norms and practices, social ranking, socio-economic status, available resources, information dissemination and monitoring tools. Therefore, there is need to harmonize what is internationally required and what is feasible to accommodate global variability. The protocols followed to ensure and evaluate farm animal welfare require regular investigation, innovation and a sustainable approach to enhance animal productivity, efficiency and product quality. Additionally, investing in animal wellbeing and health, as well as empowering communities with significant knowledge, has a potential to improve African livelihoods and contribute to food security. This review seeks to highlight the concept of animal welfare in relation to livestock and food production in African conditions.

## INTRODUCTION

The African continent could produce sufficient food to eliminate hunger and food insecurity, as well as play a major role in global food markets [1]. This is because a significant proportion of the region is conducive for various agricultural practices, which have been an integral part of its people for generations. Africa is considerably large with vast differences in geographical, climatic, cultural, historical, political and industrial situations; influencing the composition of livestock production across the region [2]. Livestock production affords an essential part of most economies; through the production of food and value-added goods, providing sustainable employment, generating cash income and security, among other things [3–5]. The Food and Agricultural Organisation (FAO), International Fund for Agricultural Development (IFAD) & World Food Program (WFP) [6] place importance on economic growth towards improving the livelihoods of people. Globally, about 40% of agricultural outputs come from livestock and this contributes to the livelihood and food security of over a billion people [7]. About 50% of African household food requirements and income is dependent on livestock farming; with the main contributing species being cattle, chickens, sheep and goats [8,9]. Furthermore, animal products can play an important role as nutrient dense food sources in human diets, as they are high in quality and readily available for absorption in the human system [10–12] and thus contribute towards food security, which is a current global challenge. Improving access to nutritious animal sourced foods is an approach that the African region can benefit from, since some of the most food insecure communities in the world are located on this continent [6]. The African Union (AU) [13] envisions a future in which agricultural production, productivity and value addition improves, enriching household, national and regional wellbeing and food security. As such, Africa shows great potential for animal agriculture [1,14,15].

Considering its wide natural resource-base [16], the continent at large can produce appreciable quantities of organic and/or free-range animal products, with a potential to supply the international market [17,18]. These products are perceived to be healthier, with higher nutritional composition and are increasingly gaining popularity and demand worldwide [19, 20]. Furthermore, this could have a positive impact on the economic growth [21,22], both at micro (individual/household) and macro (country/regional) levels. However, due to several factors (such as disease outbreaks, climate change, economic uncertainty, lack of sustainable production practices, animal welfare [AW] conditions), this potential has not been fully realised. There is stunted progress, where importing of some animal products (and other food types) outweighs exports [23]. Amongst all the contributing factors, poor AW conditions can be identified as one of the major impediments. This mostly affects smallholder farming in developing countries, an important contributor to the livelihoods of millions [4,24], owing to various limiting factors; which will be discussed later in the paper. Particularly in the African community, the AW link has not been sufficiently addressed. This review seeks to highlight the concept of AW in livestock and food production in relation to this region and how it can contribute towards mitigating food insecurity challenges.

### General overview of animal welfare and production

**Figure 1:**
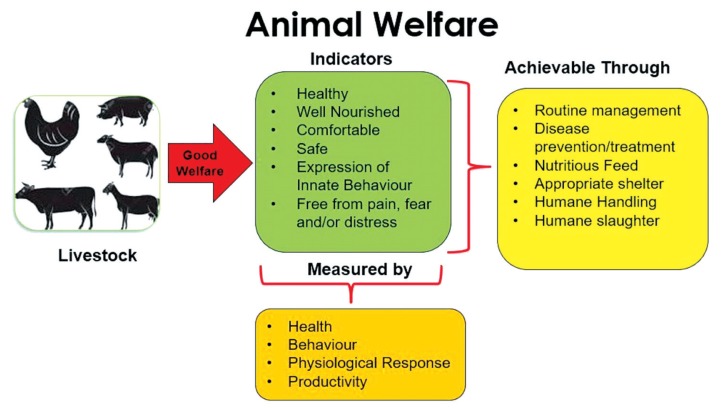
A summary of good welfare indicators, how to achieve them and how they can be quantified.

The American Veterinary Medical Association (AVMA) describes the AW concept as how an animal is coping with the conditions in which it lives; while the South African Veterinary Foundation (SAVF) describes it as a reflection of one’s concern for humane treatment of animals. Boissy et al [25] linked AW to animals being sentient and emotional beings; although some people dispute this, suggesting significant variations in the level of sensory, perceptive and cognitive awareness in different species [26]. Furthermore, AW describes how an animal is coping with its current condition, as well as its past experiences [27]. The United Kingdom (UK) Farm Animal Welfare Council (FAWC), established in 1979, developed the Five AW Freedoms, which encompass freedom from hunger and/or thirst; discomfort; pain, injury or disease; fear, stress and distress, as well as the freedom to express normal behaviour [28]. These freedoms can be achievable through specific management practices that are directly linked to each; such as access to nutritious feed and veterinary support, humane handling and slaughter, appropriate surroundings (and shelter) and management (Figure 1).

**Figure 2:**
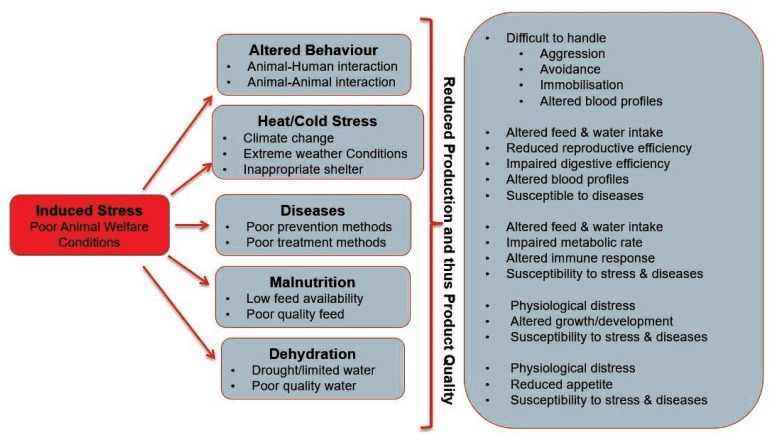
An illustration of poor animal welfare and stress indicators impacting on production and product quality.

It has been scientifically proven and reported that the ability of an animal to perform (in terms of product quality and quantity) is dependent on the conditions to which it is exposed, as well as its relationship with the stockman [29]. Losada-Espinosa et al [30] reported that in trying to cope with the presented environment, the animal’s energy is usually diverted from production to adaptation. Various food animals undergo similar stressors; however, the extent of the effects may differ. For instance, dairy cows that have been exposed to aversive handling, poor infrastructure, high/low temperatures, diseases, and/or reduced feed availability and quality may have reduced milk production [31–34]. Various meat producing animal species are generally exposed to several stressful conditions at the farm [35–37], during transportation [38–40] and slaughter at the abattoir [35,41], which may negatively affect their welfare and consequently the meat quality [32,42]. Egg quality and laying frequency is related to environmental conditions, shelter, nutrition and health [32]. Figure 2 illustrates the effect of different kinds of stress on production performance and efficiency, with ultimate consequences reflected in the quality of the end product.

### Animal welfare in African conditions

Developed countries have placed a high sense of concern for farm AW; it has been a rapidly growing area of interest over the years [15,30,43–45]. Regardless of some research-based recommendations that have been developed towards mitigating these concerns, some people generally perceive animal-based-food consumption as an inhumane act [46]. While there are international AW standards in the developed world, leading to improved management procedures, there are inherent factors that impede adoption of such in most developing regions, such as Africa. There is a need to acknowledge the geographical, climatic and systematic differences between the developed and developing worlds [15]. Some communities are uncertain about and unfamiliar with the AW concept. Furthermore, there is limited research and published literature in this area, based on African communities and practices [17]. According to Ndou et al [47], low priority is given to AW in the developing world and this can be related to traditional customs and beliefs, a lack of knowledge in animal handling and sub-standard handling facilities. Mogoa et al [48] reported that poor AW indicators in Kenya include neglect; overworking; malicious physical injury; starvation; confinement; inappropriate transportation and slaughter facilities; inhumane treatment and handling at slaughter.

Multitudes of livestock are kept by large numbers of smallholder farmers and pastoralists, producing some of the food in Africa [24,49,50]. Furthermore, small-scale farming plays an important role in the rural economy [43,51]. These are usually based in remote and/or rural areas, sometimes characterised by limited resources and access to some knowledge [5,52,53]. With limited/absence of visible governmental support on the matter, there are some NGOs that are pro AW but whose operations are mainly restricted to developed/urban areas; making them inaccessible to those areas with the greatest need [5]. Amongst other factors, which require investigation, this could be attributed to limited funds and lack of awareness about such facilities. However, with the increasing consumer scrutiny of the conditions production animals are reared in, particularly regarding their wellbeing and health, producers ought to comply. Chulayo and Muchenje [54] reported that AW is generally associated with producers, retailers and the industry, with no consumer consideration, though it may affect their attitudes towards and purchase decision of certain products. Hence, the current status regarding awareness of AW matters disqualifies the region from import and export participation with the rest of the world, as reflected in a sluggish contribution towards economic growth [14,15,47,55]. There is, therefore, a need for counteractive action from all stakeholders involved in livestock keeping [17,55]. Herrero et al [5] highlighted that livestock roles differ from one place to another. Hence other AW-affecting factors to consider in this region may include social ranking, socio-economic status, cultural norms and practices, resource availability, inadequate information dissemination strategies, as well as the lack of proper monitoring tools.

#### Cultural norms and practices

Africa is a culturally diverse region. In most African cultures, livestock is used in various traditional festivities [56]. In these instances, animals are likely to be exposed to some form of inhumane/adverse conditions, thus compromising their welfare. For instance, Lobola (bride price/dowry) cattle usually go through abrupt environmental change, sometimes sourced from various markets, regrouping/mixing with unfamiliar animals, transportation, exposure to new diseases (and sometimes death), and enclosure in a new environment. The latter can be viewed as some form of “lairaging” procedure, which is an important practice after moving animals to assist with acclimatization; while also protecting their welfare, and preventing them from straying and getting lost [57]. As alluded to by Vimiso et al [58], cattle that are put through market channels are exposed to poor AW. In addition, confinement is likely to induce some level of discomfort to the animals [17,59]; which could be escalated in an unfamiliar environment. Furthermore, some species are used for ploughing and transportation; sometimes in unfavourable conditions such as inadequate feed, water and rest [55]. In order to preserve culture and indigenous resources, allowing them to serve intended purposes, these practices are necessary. However, there is a need to sensitize participants on the potential impact of these activities on AW. Furthermore, increased handling and movement may result in additional physical demands, using up more energy [42].

Pastoral farming is another example of traditional practices found among some rural societies in East Africa [60]. Furthermore, Degen [56] reported that about 70%, 50%, and 40% of the total land in Kenya, Tanzania and Uganda, respectively, is occupied by pastoralists. In this system, the herdsman moves from one place to another with the livestock on foot, in search of feed and water [55,61]. However, there is still an un-addressed question of how these conditions influence AW. For significant environmental change, the herd travels for long distances, exposed to varying weather conditions and rangeland quality, limited water sources and possible predators (wild animals) [61–63]. Another cause of movement in some African countries is civil unrest/conflicts [64]. Cattle raiding, a cultural practice which over the years has become a more violent and criminal activity, has also been identified as a threat in this system [60,65]; threatening the safety of the herdsmen and the AW of their herds. Consequently, affected communities are increasingly forced into highly populated settlements, for the protection of their families and livestock; which however puts a strain on the already scarce natural resources, escalating poor sanitation, limited water availability and the risk of disease outbreaks [61,66]. This directly and indirectly compromises some welfare elements of both custodians and their livestock.

All food producing animals, regardless of the production system and the product, ultimately end up at slaughter once the production cycle ends. Whether it is for commercial or traditional purposes, there is an inevitable exposure of slaughter animals to multiple stressors such as handling, transportation and the slaughter process itself [15,67,68]; although the extent may differ. Traditional slaughter is normally performed in residential backyards during family gatherings or cultural events; and is generally characterised by less safe, humane and hygienic conditions [69,70]. This is sometimes translated to how local slaughterhouse employees uphold their prescribed duties in the commercial setup. For instance, some northern Nigeria slaughterhouses were noted to be inconsistent with ante-mortem and post-mortem inspection practices of slaughter animals, neglecting hygiene measurements and posing a public health threat [71]. Some African cultures perform slaughter on animals in their conscious state [69], paying little/no attention to following the suggested humane handling or slaughter practices [72]. While scientifically, animal vocalization is a stress indicator, culturally it may symbolize the success (i.e. some South African societies) or lack of it (i.e. some Namibian societies) in an event. In Kenya, chickens are often carried in non-designated modes of transport and are subjected to inhumane slaughter methods [73]. This could be a reflection of the situation in many developing countries. Most slaughter-houses in rural African communities do not measure up to appropriate standards, suggesting a need to train and monitor these abattoir personnel and properly furnish the facilities for efficient and safe operation [47,55,74,75].

#### Social ranking and socio-economic status

AW views are largely influenced by societal and individual values [55,76]. Traditionally and as far as the hierarchy of life stands, humans tend to take a higher ranking, which then influences how they view and treat everything else around them (including animals) [77]. The decision to consume animal products, such as meat, is dependent on one’s self-definition, social hierarchy and human dominance over nature [78,79]. In addition, AW perspectives are also influenced by an individual’s experiences [76] and the conditions surrounding them. For instance, in an environment with limited resources for survival, as in some parts of the African continent, it is imperative that a distinction between human and animal needs is acknowledged and prioritising humans is justifiable. People in poverty affected areas are likely to give lower priority to animals in their care; due to the existing competition for available resources [9] such as food, water, health facilities and shelter. However, investment in AW could improve production and be the very same tool that could benefit socio-economic status [17], through trade, thus contributing to household food security and income. Livestock production is an important role-player in some wellbeing indicators such as income generation, job creation and the provision of food and nutrition [62,80–82]. Furthermore, implementing good AW could contribute to improved economic growth and trade [83]. Some consumers are willing to pay more for humanely handled food animals [19,84,85]. There is need to bridge the gap between viewing intensified animal care as an economic loss and approaching it as a profitable solution.

#### Resource availability and accessibility

According to Mekuria and Aynekulu [86], the natural resource base in many developing countries has been deteriorating over time. Consequently, competition for available resources; among humans, as well as between humans and animals could be a growing challenge. Remote and/or communal areas, which practice smallholder/communal farming [87], tend to suffer the most due to minimal accessibility of these areas, limited extension support to promote sustainable land and animal management [52], as well as restricted access to affordable veterinary services. The most common production system practiced in these areas is extensive and has several AW shortcomings [17,88]. They tend to lack good soil, water and proper infrastructure, resulting in reduced production efficiency [89]. All these, in theory, have been deemed manageable with proper planning and good governance, towards efficient production. However, execution without the necessary support and facilities may still be an issue for many communities [5]. According to Grandin [90], AW inadequacy can be traced back to improper facilities, equipment and a lack of maintenance; a lack of trained stockmen and unsuitable handling. Inadequate infrastructure extends to the unavailability of electricity and proper road networks, stemming from underdevelopment in African remote/rural/pastoral areas [18,91,92]. This limits external services (extension officers, veterinarians, potential customers, feed companies, and others) from reaching the farmers, vice versa. Furthermore, it becomes costly to transport animals to various markets, due to unavailable means of transportation and the distance to travel [55,93], which also has an impact on AW. Resource-limited farmers may not be able to provide structures that shelter animals from harsh climatic conditions, leaving them exposed to discomfort, health risks and reduced productivity. Self-constructed dipping and handling facilities are seldom maintained, which could pose a threat to AW and human safety.

Livestock nutrition depends on communal grazing lands [12], which are often over utilized (overgrazing), causing major forage depletion (quality and quantity) [94,95]; thus, failure to sufficiently nourish the livestock. These are usually open fields for public use, with no proper demarcation and/or fencing to contain foraging animals. Hence in some instances livestock are found roaming around roadsides and in rural towns, posing a threat to both animal and human life. Farmers need to be familiarised with proper husbandry and veld management practices to prevent cases of veld and animal neglect. The communities are solely dependent on natural water sources [96], which are usually scarce/limited and sometimes restricted due to competition for human use. Furthermore, due to climate change, water availability has become one of the major issues in most areas, impacting agricultural activities [97]. There are concerns surrounding adaptation strategies for water storage and conservation amid water uncertainty. Water restriction reduces the animal’s appetite, increase feed utilization, as well as affect various physiological performances [98]. With resource constraints, it has become an issue of “survival of the fittest”. Mapfumo et al [99] encouraged a collaborative approach involving all stakeholders in the agricultural sector towards sustainable water use and conservation.

### Mitigation strategy 1: Regulations, information dissemination and monitoring tools

Seeing that the AW concept has economic implications [72], it is imperative that the governments assume their role in driving the directives on its functionality in the continent. There is a possibility that the legal framework and guidelines in some African countries have been developed but are not yet publicly available and therefore not well known [100]. However, to make them effective at grass-roots level, there is need to enforce them, as well as design tools to monitor implementation. According to a database compiled by Brels and Goetschel [101], only 14 (Botswana, Egypt, Kenya, Malawi, Mauritius, Namibia, Nigeria, Sao Tome and Principe, Seychelles, South Africa, Tanzania, Uganda, Zambia and Zimbabwe) out of the 54 African countries have laws against animal cruelty and legislation on AW. In South Africa for an example, DAFF [83] highlights that the current legislation that is administrated through the Animals Protection Act [102] and the Performing Animals Protection Act [103] is limited to identifying animal cruelty, but not its prevention. The World Animal Net (WAN) [104] compiled a resourceful guide as a potential starting point towards achieving “Best Practice” on AW development and implementation; focusing on i) Education and training, ii) Awareness and information, iii) Resources for Policy, Legislation and Enforcement, and iv) Resources for AW Programs. To improve animal productivity, efficiency and product quality, protocols followed to ensure and evaluate livestock welfare require investigation and innovation, as well as the development of sustainable technologies to monitor. It is of paramount importance that the whole production chain finds balance between product efficiency and AW for its continued sustainability and acceptability [46]. Furthermore, Masiga and Munyua [55] concluded that there is a need to address AW issues in Africa and the identified tools to achieve this include developing appropriate policies and regulation, as well as educating, sensitizing and encouraging involvement of communities in these issues.

Technologies to enhance AW conditions in practices such as castration [105], as well as handling and slaughter [30,106] have been improved upon and/or need to be developed over time. However, adoption has been slow in some parts of the developing world and this could be attributed to limited knowledge, means and expertise to access them. Although some of this knowledge is available on internet platforms and through specific forums; they tend to be urban centralised, restricting access for general citizens. There is need to encourage change in people’s perceptions of animals and AW; through education and better knowledge transfer. All stakeholders in the production chain must be well-informed of AW and its consequences on production [54,107]. Furthermore, among all the inspections carried out at the abattoir there is a need to incorporate ante-mortem AW assessment, to allow timely detection of possible threats to AW and to identify mitigating measures [30]. To achieve significant progress, there is need to intensify research and come up with realistic findings that are suitable and complementary to the current conditions [15]. Furthermore, it was recommended that research findings should be further translated into simple terms for the layman’s better understanding and thus implementation on the ground [15,18]. With all that being said, ensuring good quality life across different communities regardless of category, through appropriate service delivery and community development programmes for better access to resources, will improve the farmers ability to invest in and prioritize AW.

### Mitigation strategy 2: Promotion of hardy and climate resilient animals

To increase production potential and efficiency, as well as genetic gains, there is need for continuous developments in nutrition, animal health and breeding [108]. Selection of adaptable and manageable species and/or genotypes, in response to AW and climate change conditions is one possible way to achieve better herd performance. The African continent has a wide range of climatic conditions, varying from hot arid to wet tropical regions, and topography ranging from mountainous to lowlands; hence the potential for diverse livestock and animal populations to thrive in various habitats. Silanikove [98] reported that breeds which are adaptable to arid regions exhibit superior abilities to thrive under stressful conditions such as water scarcity, which has become a prominent issue. With the current state of unpredictable natural resources, mostly due to climate variability, climate resilient livestock and plant species need to be promoted to counteract the AW challenges associated with extreme drought and heat conditions [24,109]; which tend to threaten the animal’s comfortability, normal functioning and performance. Hoffman [24] also highlighted that many communities may switch to using species and breeds which are well adapted to these conditions. In line with this, Mengistu et al [110] suggested the development of simple and standardized methods of determining resilient phenotypes to identify the relationship between genetic and resilience characteristics; suitable for use in specific locations and time frames. Such developments promise better AW, with minimal resource-inputs from the caretakers. Hoffman [24] reported that locally adapted breeds, like those found in developing countries such as Africa and Asia, can survive on extensive farming systems with minimal external inputs (towards health, nutrition, shelter, etc.); while also delivering a wide range of products and services to the local community. The author also highlighted that breeds adaptable to these systems are likely to be more resilient to climate change. Hence the need is expressed to develop a more sustainable approach to livestock production, which nurtures and preserves natural resources and the environment at large. Some of the climate resilient species and/or breeds are discussed below.

Diversity among African indigenous cattle breeds, which are known to be hardy and adaptable to specific regional conditions [111], allows farmers of all production systems to choose suitable animals for efficient performance [112]. The former author further described low nutritional requirements, efficient feed utilization and commendable disease resistance as characteristics of these breeds; making them less prone to nutritional and health-related challenges. However, in some cases they have been discriminated against, especially in formal markets and other production systems such as feedlots and dairy; mostly because of their genetic characteristics and consequently their performance, which sometimes generates smaller returns compared to traditional commercial breeds. Goats are another hardy species, which can adapt and thrive in dry and unfavourable conditions; with an ability to effectively browse on woody species and to utilize low quality feed compared to other domestic ruminants [113]. The author further alluded that their unique ability to reduce metabolism allows them to efficiently use the ingested feed and water, such that they can withstand prolonged periods of insufficient food and water. Furthermore, they are a multipurpose species, providing a double protein source with health/nutritional benefits such as lean meat and milk [114–117]. However, a proper market for these products has not been established in some African countries; though some mostly consume goat meat for traditional and religious purposes [18,116], with some using goat milk to counteract malnutrition [115]. Sheep are also an important source of nutritious milk (and meat) in some parts of Africa, especially during the dry seasons [56]. This is because they flourish under extensive production systems and are adaptable to arid regions, the changing climate and increasing environmental temperatures [9]. Although they get behavioural freedom under these conditions, they may still experience other welfare challenges such as inconsistent water and feed supply, climatic variability and susceptibility to health challenges [118]. However, these can be addressed through the promotion of highly adaptable indigenous breeds and selectively breeding thermotolerant genotypes for improved productivity and reduced environmental impact [109]. Furthermore, improved management practices and monitoring strategies can be implemented as measures to enhance their welfare [119]. These could be in a form of providing locally available supplemental feed, water and shelter where and when necessary.

Indigenous chickens also possess the ability to not only rely on provided feed, but also thrive in extensive and rural setups, rummaging for natural food sources throughout the day and thus offer a more organic and affordable protein source [88,120,121]. They can be successfully reared under extensive production conditions, with minimal inputs [122, 123]; which allows them to freely express their natural behaviour. Additionally, these chickens can effectively make use of limited space and natural protein sources (such as insects) for their nourishment [88,121], providing them wider nutritional sources to satiate hunger. However, these conditions could expose them to a high risk of contracting diseases during outbreaks, due to limited biosecurity measures employed [124]. Furthermore, they take longer to reach the “acceptable” market weight and have been to have unsatisfactory egg production potential [121,125]. Furthermore, Okeno et al [126] reported that these chickens are economically viable when produced in their original genetic state and under extensive and/or semi-intensive systems. Some consumers prefer their meat compared to commercial broilers [88]. There is a need for more research towards quantifying and improving the production performance, efficiency and management of these highlighted species, leading to informed decisions for their successful inclusion in the market. Furthermore, Hoffman [24] recommended that, for all species, more research should be conducted to determine breed differences in adaptability to specific environments. Lastly, according to Shabtay [111], local breeds are biologically and economically more efficient compared to their exotic counterparts.

## CONCLUSIONS AND RECOMMENDATIONS

There is a notable potential for an efficient and respectable livestock market in Africa for African consumers and the world at large; with most of it at the hands of communal lands and small-holder farmers. However, limited knowledge of some essential concepts in production, skills and/or resources to adopt relevant procedures, capital and extension support towards maximizing on this, obscure its realization. Active State (African national governments) involvement and investment in animal wellbeing and health, as well as in empowering communities (particularly smallholder farmers) with these essentials is likely to enhance animal productivity and its revenues in the continent; improve market competency, generate income through local and international markets, as well as to improve livelihoods and contribute to food security in Africa. In addition to these efforts, commitment from farmers and stockman on implementing and following AW practices, as well as improving general management routines is of utmost importance. However, there is a need to also harmonize (through research, development and improvements in legislation, as well as incorporation of scientific knowledge in law-making for evidence-based policies) what is internationally required (AW standards) and what is feasible to bridge the gap between the developed and developing world, and thus limit hurdles that hinder participation in global trade. Furthermore, Africa should develop and nurture a strong production and market culture, governed by fitting domestic policies, accommodative of indigenous resources (breeds, knowledge and environment); in an attempt to reduce capital investments, while attaining the best profits and still promoting AW. Qualities such the reduced needs of animals from indigenous and adaptable breeds for intensive care/attention, their seamless adaptation to low levels of maintenance and management while still maintaining acceptable levels of productivity and welfare, have a potential to enhance livestock herd efficiency; especially for farmers with an inconsistent and unpredictable access to adequate resources.

## References

[1] OECD/FAO (2016). Agriculture in Sub-Saharan Africa: Prospects and challenges for the next decade. OECD-FAO Agricultural Outlook 2016–2025.

[2] New Partnership for Africa’s Development (NEPAD) (2013). African agriculture, transformation and outlook.

[3] Sere C, Steinfeld H, Groenewold J (1995). World livestock production systems: Current status, issues and trends.

[4] McDermott JJ, Staal SJ, Freeman HA, Herrero M, Van de Steeg JA (2010). Sustaining intensification of smallholder livestock systems in the tropics. Livest Sci.

[5] Herrero M, Grace D, Njuki J (2013). The roles of livestock in developing countries. Animal.

[6] Food and Agriculture Organisation (FAO), International Fund for Agricultural Development (IFAD), World Food Program (WFP) (2015). The State of Food Insecurity in the World 2015 Meeting the 2015 international hunger targets: taking stock of uneven progress.

[7] Food and Agriculture Organisation (2018). Animal production [Internet].

[8] Rust JM, Rust T (2013). Climate change and livestock production: a review with emphasis on Africa. S Afr J Anim Sci.

[9] Gowane GR, Gadekar YP, Prakash V, Kadam V, Chopra A, Prince LLL, Sejian V, Bhatta R, Gaughan J, Malik PK, Naqvi SMK, Lal R (2017). Climate Change impact on sheep production: Growth, Milk, Wool and meat. Sheep production adapting to climate change.

[10] Schönfeldt HC, Pretorius B, Hall N (2013). The impact of animal source food products on human nutrition and health. S Afr J Anim Sci.

[11] Muchenje V, Njisane YZ (2015). Why meat is important in the global battle against food insecurity [Internet].

[12] Mlambo V, Mapiye C (2015). Towards household food and nutrition security in semi-arid areas: What role for condensed tannin-rich ruminant feedstuffs?. Food Res Int.

[13] African Union (2015). Agenda 2063: The Africa we want [Internet].

[14] Scholtz MM, McManus C, Okeyo AM, Theunissen A (2011). Opportunities for beef production in developing countries of the southern hemisphere. Livest Sci.

[15] Njisane YZ, Muchenje V (2017). Farm to abattoir conditions, animal factors and their subsequent effects on cattle behavioural responses and beef quality — a review. Asian-Australas J Anim Sci.

[16] Lernoud J, Willer H, Schlatter B, Willer H, Lernoud J (2016). Africa: Current statistics. The world of organic agriculture. Statistics and emerging trends 2016.

[17] Fraser D (2008). Toward a global perspective on farm animal welfare. Appl Anim Behav Sci.

[18] Simela L, Merkel R (2008). The contribution of chevon from Africa to global meat production. Meat Sci.

[19] Harper GC, Makatouni A (2002). Consumer perception of organic food production and farm animal welfare. Br Food J.

[20] Niemeyer K, Lombard J Identifying problems and potential of the conversion to organic farming in South Africa.

[21] Lobley M, Reed M, Buttler A, Courtney P, Martyn W (2005). The impact of organic farming on the Rural Economy in England (Final Report to DEFRA). Technical Report.

[22] Gama J, Willer H, Lernoud J (2016). Latest developments in organic Agriculture in Africa. The world of organic agriculture. Statistics and emerging trends 2016.

[23] Viljoen W (2017). The face of African agriculture trade [Internet].

[24] Hoffman I (2010). Climate change and the characterization, breeding and conservation of animal genetic resources. Anim Genet.

[25] Boissy A, Aubert A, Désiré L, Greiveldinger L, Delval E, Veissier I (2011). Cognitive sciences to relate ear postures to emotions in sheep. Anim Welf.

[26] Piggins D, Phillips CJC (1998). Awareness in domesticated animals—concepts and definitions. Appl Anim Behav Sci.

[27] Boissy A, Erhard HW, Grandin T, Deesing MJ (2014). How studying interactions between animal emotions, cognition, and personality can contribute to improve animal welfare. Genetics and the behaviour of domestic animals.

[28] Farm Animal Welfare Council (2009). Farm Animal Welfare in Great Britain: Past, Present and Future [Internet].

[29] Williams JL, Richert BT, Marchant-Forde JN, Eicher SD (2012). Behavioral chances in neonatal swine after an 8-hour rest during prolonged transportation. J Anim Sci.

[30] Losada-Espinosa N, Villarroel M, María GA, Miranda-de la Lama GC (2018). Pre-slaughter cattle welfare indicators for use in commercial abattoirs with voluntary monitoring systems: a systematic review. Meat Sci.

[31] Breuer K, Hemsworth PH, Barnett JL, Matthews LR, Coleman GJ (2000). Behavioural response to humans and the productivity of commercial dairy cows. Appl Anim Behav Sci.

[32] Nardone A, Ronchi B, Lacetera N, Ranieri MS, Bernabucci U (2010). Effects of climate changes on animal production and sustainability of livestock systems. Livest Sci.

[33] Dodzi MS, Muchenje V (2011). Avoidance-related behavioural variables and their relationship to milk yield in pasture-based dairy cows. Appl Anim Behav Sci.

[34] Dodzi MS, Muchenje V (2012). Seasonal variation in time budgets and milk yield for Jersey, Friesland and crossbred cows raised in a pasture-based system. Trop Anim Health Prod.

[35] Grandin T (2003). Transferring results of behavioural research to industry to improve animal welfare on the farm, ranch and slaughter plant. Appl Anim Behav Sci.

[36] European Food Safety Authority (EFSA) (2007). Food safety aspects of different pig housing and husbandry systems. Scientific opinion of the panel on biological hazards. Eur Food Safety Auth J.

[37] Cloete JJE, Cloete SWP, Scholtz AJ, Hoffman LC (2013). Behaviour response of Namaqua Afrikaner, Dorper and South African Mutton Merino lambs towards humans. S Afr J Anim Sci.

[38] European Food Safety Authority (EFSA) Panel on Animal Health and Welfare (2011). Scientific opinion concerning the welfare of animals during transport. Eur Food Safety Auth J.

[39] Hulbert LE, Carroll JA, Burdick NC, Randel RD, Brown MS, Balloua MA (2011). Innate immune responses of temperamental and calm cattle after transportation. Vet Immunol Immunopathol.

[40] Miranda-de la Lama GC, Villarroel M, María GA (2014). Livestock transport from the perspective of the pre-slaughter logistic chain: a review. Meat Sci.

[41] Hemsworth PH, Rice M, Karlen MG (2011). Human–animal interactions at abattoirs: Relationships between handling and animal stress in sheep and cattle. Appl Anim Behav Sci.

[42] Ferguson DM, Warner RD (2008). Have we underestimated the impact of pre-slaughter stress on meat quality in ruminants?. Meat Sci.

[43] Nyika A (2009). Animal research ethics in Africa: an overview. Acta Trop.

[44] Jacques S (2014). Science and animal welfare in France and European Union: Rules, constraints, achievements. Meat Sci.

[45] Stoir S, Larsen HD, Aaslyng MD, Lykke L (2016). Improved animal welfare, the right technology and increased business. Meat Sci.

[46] Muchenje V, Mukumbo FE, Njisane YZ (2018). Meat in a sustainable food system. S Afr J Anim Sci.

[47] Ndou SP, Muchenje V, Chimonyo M (2011). Assessment and implications of animal welfare in beef production systems in developing countries. Afr J Biotechnol.

[48] Mogoa E, Wabacha J, Mbithi P, Kiama S (2005). An overview of animal welfare issues in Kenya. Kenya Vet.

[49] Statistics South Africa (2011). Census 2011 Agricultural households.

[50] National Planning Committee (NPC) (2012). National Development Plan 2030: Our future–make it work ISBN:978-0-621-40475-3 [Internet].

[51] Labarthe P, Laurent C (2013). Privatization of agricultural extension services in the EU: Towards a lack of adequate knowledge for small-scale farms?. Food Policy.

[52] Knickel K, Brunori G, Rand S, Proost J (2009). Towards a better conceptual framework for innovation processes in agriculture and rural development: from linear models to systemic approaches. J Agric Educ Ext.

[53] Logwa ET (2010). Bridging the agricultural knowledge and information divide: the case of selected telecenters and rural radio in Tanzania. EJISDC.

[54] Chulayo AY, Muchenje V (2015). A balanced perspective on animal welfare for improved meat and meat products. S Afr J Anim Sci.

[55] Masiga WN, Munyua SJM (2005). Global perspectives on animal welfare: Africa. Rev Sci Tech Off Int Epiz.

[56] Degen AA (2007). Sheep and goat milk in pastoral societies. Small Rumin Res.

[57] World Organization for Animal Health (OIE) (2011). Terrestrial Animal Health Code.

[58] Vimiso P, Muchenje V, Marume U, Chiruka R (2012). Preliminary study on consumers’ and meat traders’ perceptions of beef quality and how the beef quality is affected by animal welfare practices. Sci Res Essays.

[59] Lee C, Fisher AD, Colditz IG, Lea JM, Ferguson DM (2013). Preference of beef cattle for feedlot or pasture environments. Appl Anim Behav Sci.

[60] Gray S, Sundal M, Wiebusch B, Little MA, Leslie PW, Pike IL (2003). Cattle raiding, cultural survival, and adaptability of East African pastoralists. Curr Anthropol.

[61] Sheik-Mohamed A, Velema JP (1999). Where health care has no access: the nomadic populations of sub-Saharan Africa. Trop Med Int Health.

[62] Katjiua M, Ward D (2007). Pastoralists’ perceptions and realities of vegetation change and browse consumption in the northern Kalahari, Namibia. J Arid Environ.

[63] Din JU, Ali H, Ali A (2017). Pastoralist-predator interaction at the roof of the world: Conflict dynamics and implications for conservation. Ecol Soc.

[64] Bohra-Mishra P, Massey DS (2011). Individual decisions to migrate during civil conflict. Demography.

[65] Fleisher ML (2000). Kuria Cattle Raiders: Violence and Vigilantism on the Tanzania/Kenya Frontier.

[66] Cummings MJ, Wamala JF, Komakech IK, Malimbo M, Lukwago L (2014). Emerging and reemerging epidemic-prone diseases among settling nomadic pastoralists in Uganda. Acta Trop.

[67] Grandin T (1997). Assessment of stress during handling and transport. J Anim Sci.

[68] Hultgren J, Berg C, Karisson AH, Schiffer KJ, Algers B, Springer S, Grimm H (2018). On-farm slaughter – ethical implications and prospects. Professionals in food chains.

[69] Clottey JA (1985). Slaughter practices and techniques. Manual for the slaughter of small ruminants in developing countries.

[70] Fayemi PO, Muchenje V (2012). Meat in African context: From history to science. Afr J Biotechnol.

[71] Bello M, Lawan MK, Aluwong T, Sanusi M (2015). Management of slaughter houses in northern Nigeria and the safety of meat produced for human consumption. Food Control.

[72] Grandin T (2018). Welfare problems in cattle, pigs, and sheep that persist even though scientific research clearly shows how to prevent them. Animals.

[73] Otieno DJ, Ogutu SO Consumer willingness to pay for animal welfare attributes in a developing country context. The case of chicken in Nairobi, Kenya.

[74] Njisane YZ, Muchenje V (2013). Quantifying avoidance-related behaviour and bleeding times of sheep of different ages, sex and breeds slaughtered at a municipal and a commercial abattoir. S Afr J Anim Sci.

[75] Njisane YZ, Muchenje V (2013). Influence of Municipal abattoir conditions and animal-related factors on avoidance-related behaviour, bleeding times at slaughter and the quality of lamb meat. Asian-Australas J Anim Sci.

[76] American Veterinary Medical Association (AVMA) (2018). Animal Welfare: What is it? [Internet].

[77] Phillips CJC, Kluss K, Scanes EC, Toukhsati S (2018). Animal welfare and animal rights. Animals and human society.

[78] Allen MW, Wilson M, Ng SH, Dunne M (2000). Values and beliefs of vegetarians and omnivores. J Soc Psychol.

[79] Allen MW, Baines S (2002). Manipulating the symbolic meaning of meat to encourage greater acceptance of fruits and vegetables and less proclivity for red and white meat. Appetite.

[80] Herrero M, Thornton PK, Gerber P, Reid RS (2009). Livestock, livelihoods and the environment: understanding the trade-offs. Curr Opin Environ Sustain.

[81] Capper JL (2013). Should we reject animal source foods to save the planet? A review of the sustainability of global livestock production. S Afr J Anim Sci.

[82] Food and Agriculture Organisation (FAO) (2015). World Agriculture: Towards 2015/2030. An FAO perspective [Internet].

[83] Department of Agriculture, Forestry and Fisheries (DAFF) (2015). Animal welfare strategic implementation plan to the veterinary strategy [Internet].

[84] Miranda-de la Lama GC, Estévez-Moreno LX, Sepúlveda WS (2017). Mexican consumers’ perceptions and attitudes towards farm animal welfare and willingness to pay for welfare friendly meat products. Meat Sci.

[85] Clark B, Stewart GB, Panzone LA, Kyriazakis I, Frewer LJ (2017). Citizens, consumers and farm animal welfare: A meta-analysis of willingness-to-pay studies. Food Policy.

[86] Mekuria W, Aynekulu E (2013). Exclosure land management for restoration of the soils in degraded communal grazing lands in Northern Ethiopia. Land Degrad Develop.

[87] Mmbengwa V, Nyhodo B, Myeki L, Ngethu X, van Schalkwyk H (2015). Communal livestock farming in South Africa: Does this farming system create jobs for poverty stricken rural areas?. Sylwan.

[88] Kingori AM, Wachira AM, Tuitoek JK (2010). Indigenous chicken production in Kenya: a review. Int J Poult Sci.

[89] Department of National Treasury (DNT) (2015). Agriculture and Land. Provincial Budgets and Expenditure Review: 2010/11 - 2016/17 [Internet].

[90] Grandin T (1996). Factors that impede animal movement at slaughter plants. J Am Vet Med Assoc.

[91] Adejuwon KD (2012). The challenges of agriculture and rural development in Africa: the case of Nigeria. Int J Acad Res Prog Educ Dev.

[92] Okeno TO, Kahi AK, Peters KJ (2012). Characterization of indigenous chicken production systems in Kenya. Trop Anim Health Prod.

[93] Mahmoud HA (2008). Risky trade, resilient traders: trust and livestock marketing in northern Kenya. Africa.

[94] Nedessa B, Ali J, Nyborg I (2005). Exploring ecological and socio-economic issues for the improvement of area enclosure management: a case study from Ethiopia. Drylands Coordination Group Report.

[95] Sigwela A, Elbakidze M, Powell M, Angelstam P (2017). Defining core areas of ecological infrastructure to secure rural livelihoods in South Africa. Ecosyst Serv.

[96] Drechsel P, Olaleye A, Adeoti A, Thiombiano L, Barry B, Vohland K (2006). Adoption River and Constraints of Resource Conservation Technologies in Sub-saharan Africa [Internet]. Unpublished paper.

[97] Turral H, Burke J, Faures J (2011). Climate change, water and food security.

[98] Silanikove N (1992). Effects of water scarcity and hot environment on appetite and digestion in ruminants: a review. Livest Prod Sci.

[99] Mapfumo L, Mukumbo FE, Zhou L, Aghdasi F, Muchenje V (2014). A review on the water policy and related changes faced by resource-limited farmers in South African. J Sci Eng Technol.

[100] Nyika A (2009). Animal research ethics in Africa: an overview. Acta Trop.

[101] Brels S, Goetschel AF (2017). Database legislation: Animal legislations in the world at National level [Internet]. Global Animal Law project.

[102] (1962). Animals Protection Act. Act No. 71 of 1962 [Internet].

[103] (1935). Performing Animals Protection Act. Act No. 24 of 1935 [Internet].

[104] World Animal Net (WAN) (2017). Animal Welfare: Best Practice Resources for Animal Welfare Development & Implementation [Internet].

[105] Needham T, Lambrechts H, Hoffman LC (2017). Castration of male livestock and the potential of immunocastration to improve animal welfare and production traits. S Afr J Anim Sci.

[106] Grandin T (2017). On-farm conditions that compromise animal welfare that can be monitored at the slaughter plant. Meat Sci.

[107] Broom DM, Fraser AF (2007). Domestic animal behaviour and welfare.

[108] Thornton PK (2010). Livestock production: recent trends, future prospects. Philos Trans R Soc Lond B Biol Sci.

[109] Sejian V, Samal L, Soren NM, Sejian V, Bhatta R, Gaughan J, Malik PK, Naqvi SMK, Lal R (2017). Adaptation strategies to counter climate change effect on sheep. Sheep production adapting to climate change.

[110] Mengistu UL, Puchala R, Sahlu T, Gipson TA, Dawson LJ, Goetsch AL (2017). Conditions to evaluate differences among individual sheep and goats in resilience to high heat load index. Small Rumin Res.

[111] Shabtay A (2015). Adaptive traits of indigenous cattle breeds: the Mediterranean Baladi as a case study. Meat Sci.

[112] Strydom PE (2008). Do indigenous Southern African cattle breeds have the right genetics for commercial production of quality meat?. Meat Sci.

[113] Silanikove N (2000). The physiological basis of adaptation in goats to harsh environments. Small Rumin Res.

[114] Webb EC, Casey NH, Simela L (2005). Goat meat quality. Small Rumin Res.

[115] Haenlein GFW (2004). Goat milk in human nutrition. Small Rumin Res.

[116] Department of Agriculture, Forestry and Fisheries (DAFF) (2015). A profile of the South African goat market value chain [Internet].

[117] Garcia V, Rovira S, Boutoial K, López MB (2014). Improvements in goat milk quality: a review. Small Rumin Res.

[118] Dwyer CM (2009). Welfare of sheep: providing for welfare in an extensive environment. Small Rumin Res.

[119] Ferguson DM, Fisher A, Colditz IG, Lee C, Ferguson DM, Lee C, Fisher A (2017). Future challenges and opportunities in sheep welfare. Advances in Sheep Welfare.

[120] Bekele F, Adnoy T, Gjoen HM, Kathle J, Abebe G (2010). Production performance of dual purpose crosses of two indigenous with two exotic chicken breeds in sub-tropical environment. Int J Poult Sci.

[121] Meseret M, Solomon D, Tadelle D (2011). Marketing system, socio economic role and intra household dynamics of indigenous chicken in Gomma Wereda, Jimma Zone, Ethiopia. Livest Res Rural Dev.

[122] Mwalusanya NA, Katule AM, Mutayoba SK, Mtambo MMA (2002). Productivity of local chickens under village management conditions. Trop Anim Health Prod.

[123] Mtileni BJ, Muchadeyi FC, Maiwashe A (2009). Characterisation of production systems for indigenous chicken genetic resources of South Africa. Appl Anim Husb Rural Dev.

[124] Conan A, Goutard FL, Sorn S, Vong S (2012). Biosecurity measures for backyard poultry in developing countries: a systematic review. BMC Vet Res.

[125] Magothe T, Okeno T, Muhuyi W, Kahi A (2012). Indigenous chicken production in Kenya: I. Current status. World’s Poult Sci J.

[126] Okeno TO, Magothe TM, Kahi AK, Peters KJ (2012). Breeding objectives for indigenous chicken: Model development and application to different production systems. Trop Anim Health Prod.

